# Commentary: Efficacy and safety of acupuncture on symptomatic improvement in primary Sjögren's syndrome: A randomized controlled trial

**DOI:** 10.3389/fmed.2022.1098862

**Published:** 2023-01-04

**Authors:** Hsin-Yuan Chen, Jin-Huang Wu, Hong-Chun Lin, Yu-Ting Su, Chien-Ming Yen, Ching-Mao Chang

**Affiliations:** ^1^Center for Traditional Medicine, Taipei Veterans General Hospital, Taipei, Taiwan; ^2^School of Post-baccalaureate Chinese Medicine, China Medical University, Taichung, Taiwan; ^3^Institute of Traditional Medicine, National Yang Ming Chiao Tung University, Taipei, Taiwan; ^4^Faculty of Medicine, National Yang Ming Chiao Tung University, Taipei, Taiwan

**Keywords:** acupuncture, Sjögren's syndrome, traditional Chinese medicine, sham effects, auto-immune

In the prospective study titled “Efficacy and safety of acupuncture on symptomatic improvement in primary Sjögren's syndrome: a randomized controlled trial” (1), Prof. Jiang and colleagues evaluated acupuncture treatment of the main symptoms of primary Sjögren's syndrome (pSS), specifically dryness, pain, and fatigue. Their results suggested that acupuncture did not improve these symptoms more than a placebo did. However, in contrast to pharmacological interventions (2–6), acupuncture therapies depend on procedural expertise and are often complex and multifaceted.

Moreover, prior clinical and basic scientific literature support the possibility of specific effects being generated by sham acupuncture (7). It is therefore possible that acupuncture's reported failure to improve the main symptoms of pSS was related to the authors' use of the same acupoints and manipulations in their real and sham acupuncture groups; and that the reported lack of inter-group differences could have been influenced, at least in part, by the ostensibly sham condition having real effects, or at any rate, strong placebo effects. To avoid such a possibility, recent acupuncture trials have used non-acupoint locations for sham acupuncture. Zhao et al. (8), for example, adopted such an approach and found that true acupuncture was more closely associated with long-term reductions in migraine recurrence that either sham acupuncture or being placed on a waiting list.

According to Zhang et al.'s study published in 2022 in the *BMJ* (9), trialists should carefully consider the desirability of sham conditions that might lead to underestimation of acupuncture treatments' clinical effects. The lack of a placebo effect in prior acupuncture trials raised concerns about the integrity of the sham blinding, unintentional crossover in the sham treatment groups, and/or a physiological effect of the sham acupuncture (8). Hershman et al., to address prior concerns about these possible sham effects by ascertaining whether psychological effects are in play, designed a study with a waitlist control group whose members received no therapeutic intervention (10). In such a control group, the placebo effects arising from the acupuncturist-patient relationship and patients' expectations about the benefits of acupuncture may be zero, unlike those experienced by a sham acupuncture group (8).

Traditional Chinese medicine holds that an acupoint is a specific point on the body's surface that is inactive when the body is in a healthy condition, but which can be activated by certain stimuli. Brain activation associated with acupuncture stimulation has been found to be influenced by enhanced bodily awareness and bodily attention around the acupoints (11); and it has been shown that the spatial configurations of De-qi sensations in response to tactile stimulation can be influenced by visual bio-signal information. This suggests that information on physiological responses to acupuncture stimulations can change participants' expectations of how somatic sensations are perceived, as well as their expectations of how they will interpret the stimulations themselves. Moreover, expectations can be formed *via* various mechanisms, including verbal suggestions and conditioning (12). Therefore, when Prof. Jiang and colleagues informed their subjects that they would undergo “a new method of acupuncture”, it could have affected the latter's expectations.

The meaning of *de-qi* was acupuncture-related sensations of four types, i.e., soreness, numbness, fullness/distention and heaviness. However, factors related to understanding *de-qi*, such as its mechanisms, its related organs, its biological parameters, and even its relationship with acupuncture's efficacy remain unclear. According to Liang et al.'s study published in 2013 (13), volunteers could not distinguish between the *de-qi* feelings induced by real and sham needles, irrespective of whether the shift was from real to sham or vice versa; and a non-invasive placebo acupuncture device could simulate the *de-qi* sensation as effectively as real acupuncture. In another review (14), we found that real and sham acupuncture modulated different brain regions/networks, which may suggest that different mechanisms underlie these two techniques (15, 16). Hence, the choice of sham acupoints could be an important factor in sham acupuncture's efficacy.

In conclusion, due to the use of the same acupoints across both the real and sham acupuncture groups, a variety of stimuli could have had similar responses; acupuncture stimulations also can affect participants' expectations; and the lack of a waiting-list group is concerning. Hence, we are not convinced by Prof. Jiang and colleagues' results that acupuncture treatment had no value in improving pSS symptoms.

## Author contributions

H-YC, J-HW, H-CL, Y-TS, C-MY, and C-MC were responsible for the study concept and design, modification of the study design, and review and interpretation of the data. H-YC, J-HW, and H-CL were responsible for drafting the manuscript. C-MY and C-MC made modifications to the study design and revised the manuscript. H-YC, J-HW, H-CL, and Y-TS contributed to the collection and analysis of data. H-CL, Y-TS, C-MY, and C-MC contributed to the interpretation of the data and revised the manuscript. All authors read and approved the final manuscript.
